# Serum 25-hydroxy vitamin D and the risk of low muscle mass in young and middle-aged Korean adults

**DOI:** 10.1530/EJE-21-1229

**Published:** 2022-02-11

**Authors:** Yejin Kim, Yoosoo Chang, Seungho Ryu, In Young Cho, Min-Jung Kwon, Sarah H Wild, Christopher D Byrne

**Affiliations:** 1 Center for Cohort Studies, Total Healthcare Center; 2 Department of Occupational and Environmental Medicine, Kangbuk Samsung Hospital, Sungkyunkwan University School of Medicine, Seoul, Republic of Korea; 3 Department of Clinical Research Design & Evaluation, SAIHST, Sungkyunkwan University, Seoul, Republic of Korea; 4 Department of Family Medicine, Kangbuk Samsung Hospital, Sungkyunkwan University School of Medicine, Seoul, Republic of Korea; 5 Department of Laboratory Medicine, Kangbuk Samsung Hospital, Sungkyunkwan University School of Medicine, Seoul, Republic of Korea; 6 Usher Institute, University of Edinburgh, Edinburgh, UK; 7 Nutrition and Metabolism, Faculty of Medicine, University of Southampton, Southampton, UK; 8 National Institute for Health Research Southampton Biomedical Research Centre, University Hospital Southampton, Southampton, UK

## Abstract

**Objective:**

Despite the known benefit of vitamin D in reducing sarcopenia risk in older adults, its effect against muscle loss in the young population is unknown. We aimed to examine the association of serum 25-hydroxy vitamin D [25(OH)D] level and its changes over time with the risk of incident low muscle mass (LMM) in young and middle-aged adults.

**Design:**

This study is a cohort study.

**Methods:**

The study included Korean adults (median age: 36.9 years) without LMM at baseline followed up for a median of 3.9 years (maximum: 7.3 years). LMM was defined as the appendicular skeletal muscle (ASM) mass by body weight (ASM/weight) of 1 s.d. below the sex-specific mean for the young reference group. Cox proportional hazard models were used to estimate hazard ratios (HRs) with 95% CIs.

**Results:**

Of the 192,908 individuals without LMM at baseline, 19,526 developed LMM. After adjusting for potential confounders, the multivariable-adjusted HRs (95% CIs) for incident LMM comparing 25(OH)D levels of 25–<50, 50–<75, and ≥75 nmol/L to 25(OH)D <25 nmol/L were 0.93 (0.90–0.97), 0.85 (0.81–0.89), and 0.77 (0.71–0.83), respectively. The inverse association of 25(OH)D with incident LMM was consistently observed in young (aged <40 years) and older individuals (aged ≥40 years). Individuals with increased 25(OH)D levels (<50–≥50 nmol/L) or persistently adequate 25(OH)D levels (≥50 nmol/L) between baseline and follow-up visit had a lower risk of incident LMM than those with persistently low 25(OH)D levels.

**Conclusions:**

Maintaining sufficient serum 25(OH)D could prevent unfavourable changes in muscle mass in both young and middle-aged Korean adults.

## Introduction

Sarcopenia is characterized by a progressive decline in skeletal muscle mass and muscle strength (1) and represents a major public health concern in older adults. Sarcopenia can lead to serious health consequences that impair the quality of life and pose a considerable burden on healthcare systems (1, 2). Although sarcopenia is more commonly associated with older ages, there is growing recognition that sarcopenia also occurs early in life, partly due to increased sedentary lifestyle and physical inactivity in the modern young population (1, 3, 4). However, risk or protective factors associated with sarcopenia or muscle loss in younger individuals have not been adequately addressed and remain largely unknown.

Beyond its widely recognized effects on bone health, vitamin D is known to affect skeletal muscle via vitamin D receptors (VDRs) (5). The link between low serum 25-hydroxyvitamin D [25(OH)D] levels, a reliable marker of vitamin D status, and the risk of sarcopenia in older individuals is well established (6, 7). Several cross-sectional studies explored the relationship between vitamin D deficiency and muscle mass, but the results were conflicting (8, 9, 10, 11). Also, findings from small randomized–controlled trials (RCTs) showed no benefit of vitamin D supplementation on muscle mass gain in young individuals (12, 13). With the lack of large and high-quality studies, it remains unclear whether vitamin D has any protective effect against low muscle mass (LMM) development in young people. In addition, no studies have yet to evaluate the effect of changes in serum 25(OH)D levels overtime on the risk of developing LMM.

Thus, we examined the association of serum 25(OH)D level and its changes over time with the risk of incident LMM in young and middle-aged adults without LMM at baseline.

## Subjects and methods

### Study participants

**Figure 1:**
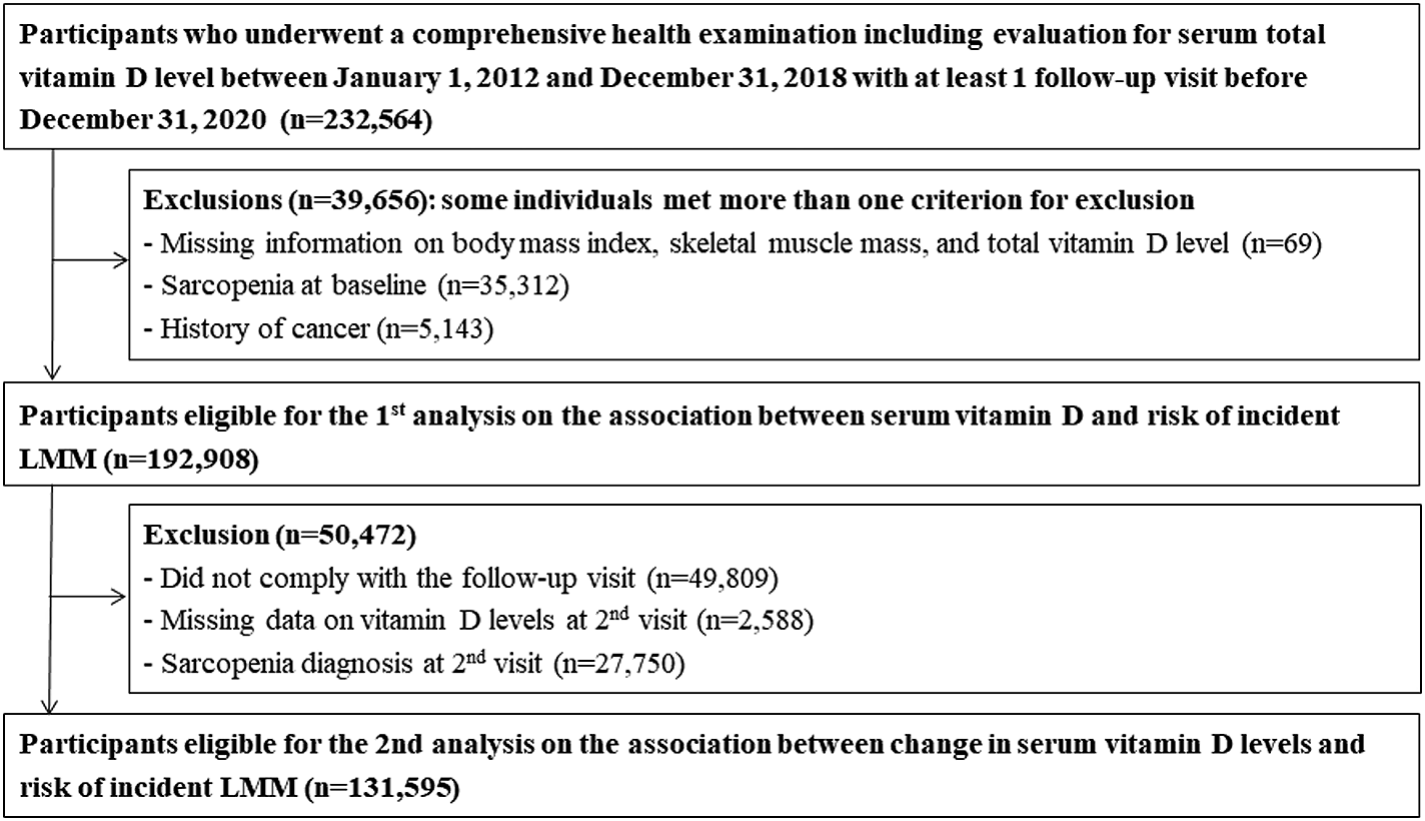
Selection of study participants.

The Kangbuk Samsung Health Study is a cohort study of Korean men and women aged ≥18 years who participated in comprehensive health examinations every 1–2 years at Kangbuk Samsung Hospital Total Healthcare Center in Seoul and Suwon, South Korea, as previously described (14). The present cohort study included participants who underwent comprehensive health examinations between January 2012 and December 2018. From 2012, data on appendicular skeletal muscle mass and serum 25(OH)D levels were available, and all participants had at least one follow-up visit between recruitment and 31 December 2020 (*n* = 232,564 participants). A total of 39,656 participants were excluded in a two-step selection process (Fig. 1) (see Supplementary Material for detailed exclusion criteria, see section on supplementary materials given at the end of this article).

This study was approved by the Institutional Review Board of Kangbuk Samsung Hospital (IRB no. KBSMC 2021-09-032), which waived the requirement for informed consent because de-identified retrospective data routinely collected during health screenings were used.

### Measurements

At baseline and follow-up visits, information on demographic factors, lifestyle factors such as physical activity, medical history, and medication use was obtained using standardized, self-administered questionnaires (14).

Physical activity was assessed using the short form of the validated Korean version of the International Physical Activity Questionnaire (15). According to this questionnaire’s results, the participants were categorized as being inactive, being minimally active, or engaging in health-enhancing physical activity (HEPA). HEPA was defined as follows: (i) vigorous activity for ≥3 days/week with ≥1500 accumulated metabolic equivalent (MET)-min/week or (ii) a combination of walking and moderate- or vigorous-intensity activities for 7 days totalling to ≥3000 MET-min/week.

Blood pressure, height, weight, and body composition measurements were performed by trained nurses. A multi-frequency bio-impedance analyzer (BIA) with eight-point tactile electrodes (InBody 720; Biospace Inc., Seoul, Korea) was used to measure body composition including lean body mass of individuals’ limbs, appendicular skeletal muscle mass (ASM), and fat mass. The body composition using the BIA (InBody720) was reliable in men and women as indicated by high intraclass correlation coefficient for measures of body composition of ≥0.98 including skeletal muscle mass (16, 17). The BIA technique was validated for the assessment of body composition, showing a good correlation with dual-energy X-ray absorptiometry (DEXA), and applied to estimate ASM in various populations (18, 19, 20, 21, 22, 23). The InBody720 demonstrated a strong correlation with DXA in ASM (Pearson correlation coefficients 0.944 and 0.903, and s.e. of estimate 1.051 kg and 0.927 kg in men and women, respectively) (24). Obesity was defined as BMI ≥ 25 kg/m^2^, which is the cut-off value for diagnosing obesity in Asians (25).

Blood specimens were collected after a fasting period of at least 10 h, and fasting blood tests evaluated glycemic parameters, lipid profiles, liver enzyme levels, and high-sensitivity C-reactive protein (hsCRP) levels (26). Insulin resistance was estimated using the homeostatic model assessment–insulin resistance (HOMA-IR) equation as follows: fasting blood insulin (uU/mL) × fasting blood glucose (mmol/L)/22.5; the cut-off value of 2.5 was used (27). Hypertension was defined as a systolic blood pressure ≥ 140 mmHg, diastolic blood pressure ≥ 90 mmHg, or current use of blood pressure-lowering medication. Diabetes mellitus was defined as a fasting serum glucose level ≥ 126 mg/dL, haemoglobin A1c ≥ 6.5%, or current use of anti-diabetic medications or insulin.

Skeletal muscle mass index (SMI) was calculated using BIA as SMI (%) = appendicular skeletal muscle mass (kg)/body weight (kg) × 100, according to the methods by Janssen et al (28). Normal SMI was defined as an SMI higher than −1 s.d. below the sex-specific mean of young reference adults (aged 20–39 years) (28). Among young adults (20–39 years old) in this study population, the mean (s.d.) of SMI was 32.9 % (2.7) for men and 28.6 % (2.5) for women. Class I LMM was defined as an SMI within −1 to −2 s.d. below the mean values of young adults, and class II LMM was defined as SMI below −2 s.d. below the mean values of young adults (28). Because early detection of muscle mass loss in young adults is important, incident LMM was defined according to class I LMM development.

To assess serum 25(OH)D status, total 25(OH)D levels, including 25(OH)D_2_ and 25(OH)D_3_, were measured with a competitive immunoassay using an Elecsys Vitamin D Total assay on the Modular E170 (Roche Diagnostics) until April 2015 and Cobas e801 (Roche Diagnostics) thereafter. Total 25(OH)D measurement using the Elecsys vitamin D total assay demonstrated acceptable performance compared to using liquid chromatography-tandem mass spectrometry, the reference standard for 25(OH)D measurement (29, 30). When the analytical performance for precision was evaluated according to CLSI-EP15-A2 guidelines, the inter-assay coefficients of variation for quality control specimens of lower and higher levels of total 25(OH)D were 2.01–5.94 and 2.69–5.03%, respectively, during the study period. The detection limit was determined according to the CLSI EP17-A2 guidelines and was reported to be <3 ng/mL (<7.5 nmol/L). Serum 25(OH)D levels were categorized as <10, 10–<20, 20–<30, and ≥30 ng/mL (for conversion to SI units: ng/mL × 2.5 = nmol/L; e.g. <25, 25–<50, 50–<75, and ≥75 nmol/L) (31, 32). Despite some controversy, serum 25(OH)D level >20 ng/mL (>50 nmol/L) is considered sufficient for skeletal health in the healthy general population (33, 34). Therefore, the change in 25(OH)D status from baseline to the second visit was analysed in the following four groups based on the presence/absence of insufficient serum 25(OH)D (defined as serum 25(OH)D level <20 ng/mL (50 nmol/L)): (i) insufficient 25(OH)D level at baseline and follow-up (persistently low); (ii) insufficient 25(OH)D level at baseline but no insufficiency at follow-up (increased); (iii) no insufficiency at baseline but insufficiency at follow-up (decreased); and (iv) no 25(OH)D insufficiency at baseline and follow-up (persistently adequate).

### Statistical analysies

Baseline characteristics of the study participants are presented according to the 25(OH)D categories mentioned above. To determine linear trends, the median values of each category were included in each model.

The primary outcome was the development of incident LMM. Each participant was followed from the baseline visit until either the occurrence of incident LMM or the last health examination conducted through the end of 2020, whichever occurred first. The incidence rates were calculated as the number of incident cases divided by person-years of follow-up. Cox proportional hazard models were used to estimate the hazard ratios (HRs) with 95% CIs for incident LMM in each 25(OH)D category compared with the reference category.

We used three models with progressive adjustment to control for potential confounders. The first model (Model 1) was adjusted for age, sex, centre, year of screening, alcohol consumption, smoking, physical activity, total energy intake, education level, medication for hypertension, medication for diabetes, multivitamin supplementation, calcium supplementation, and season. Given the potential impact of obesity on the relationship between serum 25(OH)D levels and sarcopenia (10), the model was additionally adjusted for BMI (Model 2). Alternatively, analyses were performed with adjustment for waist circumference instead of BMI. To evaluate the effects of changes in serum 25(OH)D levels and other covariates during the follow-up period, we performed additional analyses by introducing serum 25(OH)D levels and other factors as time-varying covariates in the models.

We assessed the proportional hazards assumption by examining graphs of estimated log (−log) survival. Pre-defined subgroup analyses were performed after stratifying by age (<40 vs ≥40 years), current smoking status (no vs yes), alcohol intake (<20 vs ≥20 g/day), HEPA (no vs yes), obesity defined using the specific criteria for Asians (BMI<25 kg/m^2^ vs ≥25 kg/m^2^ (25, 35)), hypertension (no vs yes), diabetes (no vs yes), HOMA-IR (<2.5 vs ≥2.5), and hsCRP (<1.0 mg/L vs ≥1.0 mg/L). The interactions according to subgroup characteristics were tested using likelihood ratio tests that compared models with and without multiplicative interaction terms. As a sensitivity analysis, the association between serum 25(OH)D levels and incident LMM was tested using LMM defined as SMI less than −2 s.d. below the mean values of young adults.

STATA version 16.0 (Stata Corp.) was used for data analysis. All *P*-values were two-tailed, and *P*-values <0.05 were considered statistically significant.

## Results

The median age of the participants was 36.9 years (interquartile range, 32.4–41.8 years), and 44.5% of patients were females. At baseline, the proportions of participants with 25(OH)D levels <25, 25–<50, 50–<75, and ≥75 nmol/L were 16.2, 56.6, 21.9, and 5.3%, respectively (Table 1). Serum 25(OH)D levels were positively associated with age, alcohol intake, physical activity, education level, medication use for hyperlipidaemia, and use of multivitamin, vitamin D, and/or calcium supplements (Table 1). Baseline characteristics of the study participants are also presented according to 25(OH)D levels at baseline and subsequent visits (Supplementary Table 1).

**Table 1:** Estimated^a^ mean and adjusted^a^ proportions of baseline characteristics by serum 25(OH)D levels among participants (*n*  = 192,908).

Characteristics	Serum 25(OH)D levels (nmol/L)				*P*
	<25	25–<50	50–<75	≥75	
Participants, *n*	31,224	109,197	42,200	10,287	
Age (years)	37.3 (37.2–37.4)	37.5 (37.5–37.6)	38.6 (38.5–38.6)	40.0 (39.8–40.1)	<0.001
Male (%)	35.27 (34.74–35.80)	56.68 (56.38–56.97)	66.27 (65.81–66.72)	59.94 (58.99–60.89)	<0.001
Alcohol intake (%)^b^	14.50 (14.05–14.94)	18.78 (18.55–19.00)	23.31 (22.94–23.68)	25.69 (24.89–26.49)	<0.001
Current smoker (%)	15.10 (14.63–15.57)	16.75 (16.54–16.95)	19.28 (18.96–19.61)	20.09 (19.39–20.78)	<0.001
HEPA (%)	11.06 (10.70–11.42)	13.58 (13.38–13.79)	17.00 (16.64–17.36)	19.73 (18.96–20.50)	<0.001
Education level (%)^c^	82.92 (82.51–83.33)	84.95 (84.74–85.16)	85.33 (84.98–85.67)	85.16 (84.48–85.84)	<0.001
History of diabetes (%)	1.61 (1.44–1.77)	1.83 (1.75–1.91)	1.83 (1.71–1.94)	1.76 (1.55–1.96)	0.336
History of hypertension (%)	5.79 (5.49–6.10)	6.02 (5.88–6.16)	6.19 (5.99–6.40)	6.40 (5.99–6.81)	0.006
History of CVD (%)	0.81 (0.70–0.93)	0.84 (0.78–0.89)	0.80 (0.72–0.88)	0.86 (0.71–1.01)	0.986
Anti-lipid medication use (%)	1.98 (1.80–2.16)	1.80 (1.72–1.88)	1.73 (1.62–1.84)	1.97 (1.76–2.18)	0.507
Multivitamin supplement (%)	3.49 (3.29–3.69)	6.05 (5.91–6.19)	10.42 (10.13–10.72)	14.95 (14.28–15.62)	<0.001
Vitamin D supplement (%)	0.21 (0.17–0.26)	0.62 (0.57–0.66)	1.74 (1.61–1.87)	4.79 (4.38–5.19)	<0.001
Calcium supplement (%)	0.20 (0.16–0.25)	0.38 (0.35–0.42)	0.98 (0.87–1.08)	2.12 (1.85–2.39)	<0.001
Obesity (%)^d^	19.42 (18.94–19.91)	21.49 (21.26–21.72)	22.49 (22.14–22.84)	19.22 (18.53–19.92)	<0.001
BMI (kg/m^2^)	22.5 (22.5–22.6)	22.7 (22.7–22.7)	22.8 (22.8–22.8)	22.5 (22.4–22.5)	<0.001
SBP (mmHg)	107.0 (106.9–107.1)	107.5 (107.5–107.6)	107.8 (107.7–107.9)	108.0 (107.8–108.2)	<0.001
DBP (mmHg)	68.8 (68.7–68.9)	69.3 (69.2–69.3)	69.5 (69.5–69.6)	69.2 (69.1–69.4)	<0.001
Glucose (mg/dL)	93.2 (93.1–93.4)	93.5 (93.5–93.6)	93.6 (93.5–93.7)	93.1 (92.9–93.3)	0.191
Total cholesterol (mg/dL)	188.0 (187.6–188.3)	191.1 (190.9–191.3)	191.8 (191.5–192.1)	190.6 (190.0–191.3)	<0.001
GGT (U/L)	26.4 (26.0–26.8)	28.1 (27.9–28.3)	30.3 (30.0–30.6)	29.9 (29.3–30.6)	<0.001
ALT (U/L)	21.5 (21.3–21.7)	22.0 (21.9–22.1)	22.5 (22.3–22.6)	22.6 (22.3–23.0)	<0.001
HOMA-IR	1.45 (1.43–1.46)	1.44 (1.44–1.45)	1.43 (1.42–1.44)	1.33 (1.32–1.35)	<0.001
hsCRP (mg/L)	0.90 (0.87–0.93)	0.91 (0.89–0.93)	0.94 (0.91–0.97)	0.94 (0.88–1.00)	<0.001
Total energy intake (kcal/d)^e, f^	1,450.6 (1,442.2–1,458.9)	1,446.5 (1,442.2–1,450.9)	1,434.7 (1,427.6–1,441.7)	1,411.7 (1,397.4–1,426.1)	<0.001

^a^Adjusted for age and sex; ^b^≥20 g/day;^ c^≥College graduate;^ d^BMI ≥ 25 kg/m^2^;^ e^Among 132,466 participants with plausible estimated energy intake levels (within 3 s.d. from the log-transformed mean energy intake); ^f^1 kcal equals to 4185.8 J.

ALT, alanine aminotransferase; CVD, cardiovascular disease; DBP, diastolic blood pressure; GGT, gamma-glutamyltransferase; HEPA, health-enhancing physically active; hsCRP, high-sensitivity C-reactive protein; HOMA-IR, homeostasis model assessment of insulin resistance; SBP, systolic blood pressure

**Figure 2:**
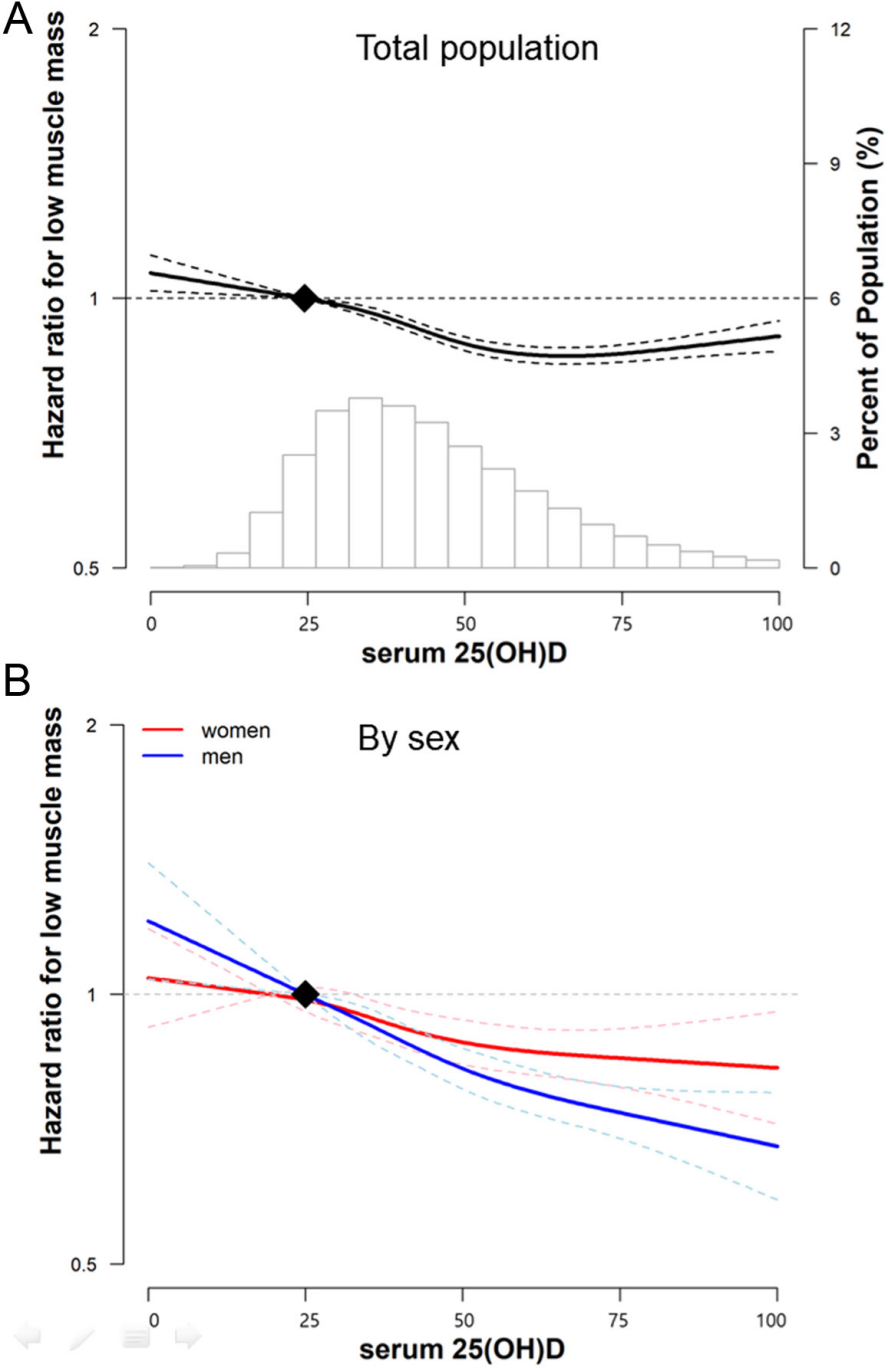
Multivariable-adjusted hazard ratios for the development of low muscle mass in the total population (A) and sex-specific (B). Curves represent adjusted hazard ratios for low muscle mass based on restricted cubic splines with knots at the 5th, 35th, 65th, and 95th percentiles of serum 25(OH)D distribution. Models were adjusted for age, sex (only for total), centre, year of screening examination, alcohol consumption, smoking, physical activity, total energy intake, education level, ongoing medication for hypertension and/or diabetes, multivitamin and/or calcium supplementation, season, and BMI. A full colour version of this figure is available at https://doi.org/10.1530/EJE-21-1229.

Within 720,713.2 person-years of follow-up (median, 3.9 years; interquartile range, 2.1–5.0 years; maximum, 7.3 years), 19,526 participants developed LMM (incidence rate, 27.1 per 1000 person-years) (Table 2). Overall, baseline 25(OH)D levels were inversely associated with the risk of incident LMM. After adjusting for age, sex, physical activity, and other potential confounders (model 1), HRs (95% CI) for incident LMM at baseline 25(OH)D levels of 25–<50, 50–<75, and ≥75 nmol/L (compared to the reference, <25 nmol/L) were 0.93 (0.90–0.97), 0.83 (0.79–0.88), and 0.67 (0.62–0.73), respectively. Further adjustment for either BMI or waist circumference attenuated the association, which remained significant (Table 2, model 2, and Supplementary Table 2). The inverse association was consistently observed in men and women but with a slightly stronger effect in men than in women (*P* for interaction = 0.025). The association between 25(OH) level and incident LMM became stronger in time-dependent analyses than in the original analyses. Corresponding HRs (95% CI) comparing 25(OH)D levels of 25–<50, 50–<75, and ≥75–<25 nmol/L were 0.79 (0.76–0.83), 0.65 (0.62–0.68), and 0.52 (0.48–0.56), respectively. In spline regression models, the LMM risk decreased across the range of the 25(OH) level in both men and women (Fig. 2). Similar results were observed in a sensitivity analysis using LMM defined as SMI less than −2 s.d. below the mean values of young adults (Supplementary Table 3).

**Table 2:** Development of low muscle mass according to serum 25(OH)D levels among participants at baseline (*n*  = 192,908). *P* = 0.025 for the overall interaction between sex and serum 25(OH)D levels for incident low muscle mass (multivariable-adjusted model 2).

25(OH)D levels (nmol/L)	Person-years	Incident cases	Incidence density (/10^3^ PY)	Age-adjusted HR (95% CI)	Multivariable-adjusted HR^a^ (95% CI)		HR (95% CI)^b^ in a model with time-dependent variables
					Model 1	Model 2	
Total (*n* = 192,908)							
<25	120,174.0	3539	29.4	1.00 (reference)	1.00 (reference)	1.00 (reference)	1.00 (reference)
25–<50	412,350.5	11,324	27.5	0.92 (0.89–0.96)	0.93 (0.90–0.97)	0.93 (0.90–0.97)	0.79 (0.76–0.83)
50–<75	154,073.9	3914	25.4	0.83 (0.79–0.87)	0.83 (0.79–0.88)	0.85 (0.81–0.89)	0.65 (0.62–0.68)
≥75	34,114.8	749	22.0	0.68 (0.63–0.74)	0.67 (0.62–0.73)	0.77 (0.71–0.83)	0.52 (0.48–0.56)
*P*				<0.001	<0.001	<0.001	<0.001
Women (*n* = 85,898)							
<25	78,608.8	2499	31.8	1.00 (reference)	1.00 (reference)	1.00 (reference)	1.00 (reference)
25–<50	175,005.9	5428	31.0	0.97 (0.92–1.01)	0.94 (0.90–0.99)	0.96 (0.91–1.00)	0.81 (0.77–0.86)
50–<75	47,255.6	1359	28.8	0.87 (0.81–0.92)	0.84 (0.78–0.90)	0.90 (0.84–0.97)	0.67 (0.63–0.72)
≥75	11,863.1	291	24.5	0.69 (0.61–0.78)	0.67 (0.59–0.76)	0.82 (0.72–0.92)	0.56 (0.50–0.62)
*P*				<0.001	<0.001	<0.001	<0.001
Men (*n* = 107,010)							
<25	41,565.2	1040	25.0	1.00 (reference)	1.00 (reference)	1.00 (reference)	1.00 (reference)
25–<50	237,344.6	5896	24.8	0.98 (0.92–1.05)	0.92 (0.86–0.98)	0.88 (0.82–0.94)	0.74 (0.69–0.80)
50–<75	106,818.3	2555	23.9	0.92 (0.86–0.99)	0.82 (0.77–0.89)	0.78 (0.72–0.84)	0.61 (0.56–0.65)
≥75	22,251.7	458	20.6	0.76 (0.68–0.84)	0.67 (0.60–0.75)	0.71 (0.63–0.79)	0.48 (0.43–0.53)
*P*				<0.001	<0.001	<0.001	<0.001

^a^Estimated using Cox proportional hazard models. Multivariable model 1 was adjusted for age, sex (only for total), centre, year of screening examination, alcohol consumption, smoking, physical activity, total energy intake, education level, medication for hypertension, medication for diabetes, multivitamin supplement use, calcium supplement use, and season; model 2: model 1 plus adjustment for BMI; ^b^Estimated using Cox proportional hazard models with categories of serum 25(OH)D levels, smoking, alcohol consumption, physical activity, total energy intake, medication for hypertension, medication for diabetes, multivitamin supplement use, calcium supplement use, season, and BMI as time-dependent variables and baseline age, sex (only for total), centre, year of screening examination, and education level as time-fixed variables.

HR, hazards ratio; PY, person-year.

Changes in 25(OH)D levels from baseline to follow-up were significantly associated with the risk of incident LMM without any significant interaction by sex (*P* for interaction = 0.326) (Table 3). The multivariable-adjusted HRs (95% CI) for the ‘decreased’, ‘increased’, and ‘persistently adequate’ groups vs the ‘persistently low’ group for LMM development were 0.84 (0.77–0.92), 0.85 (0.79–0.91), and 0.81 (0.75–0.87), respectively (model 2). The significant associations persisted after serum 25(OH)D levels and other confounders were considered time-varying variables.

**Table 3:** LMM development by the changes in serum 25(OH)D level from baseline to subsequent visit (*n*  = 131,595). *P* = 0.326 for the overall interaction between sex and sex and serum 25(OH)D levels for incident low muscle mass (multivariable-adjusted model 2).

25(OH)D levels (nmol/L)		Person-years	Incident cases	Incidence density (/10^3^ PY)	Age- and sex-adjusted HR (95% CI)	Multivariable-adjusted HR^a^ (95% CI)		HR (95% CI)^b^ in a model with time-dependent variables
Visit 1	Visit 2					Model 1	Model 2	
Total								
<50	<50	212,785.0	4157	19.5	1.00 (reference)	1.00 (reference)	1.00 (reference)	1.00 (reference)
≥50	<50	33,319.2	556	16.7	0.85 (0.78–0.93)	0.85 (0.78–0.93)	0.84 (0.77–0.92)	0.86 (0.78–0.94)
<50	≥50	63,741.6	1001	15.7	0.80 (0.75–0.86)	0.85 (0.80–0.92)	0.85 (0.79–0.91)	0.87 (0.81–0.93)
≥50	≥50	60,559.7	956	15.8	0.79 (0.74–0.85)	0.81 (0.75–0.87)	0.81 (0.75–0.87)	0.84 (0.78–0.90)
Women								
<50	<50	101,903.1	2262	22.2	1.00 (reference)	1.00 (reference)	1.00 (reference)	1.00 (reference)
≥50	<50	10,964.2	201	18.3	0.83 (0.72–0.95)	0.81 (0.70–0.93)	0.88 (0.76–1.02)	0.87 (0.75–1.01)
<50	≥50	25,365.8	410	16.2	0.73 (0.66–0.81)	0.76 (0.69–0.85)	0.80 (0.72–0.89)	0.84 (0.75–0.93)
≥50	≥50	15,201.1	257	16.9	0.75 (0.66–0.85)	0.75 (0.66–0.86)	0.83 (0.72–0.94)	0.89 (0.78–1.01)
Men								
<50	<50	110,881.9	1895	17.1	1.00 (reference)	1.00 (reference)	1.00 (reference)	1.00 (reference)
≥50	<50	22,355.0	355	15.9	0.93 (0.83–1.04)	0.84 (0.75–0.94)	0.83 (0.74–0.93)	0.85 (0.76–0.95)
<50	≥50	38,375.8	591	15.4	0.90 (0.82–0.99)	0.93 (0.85–1.03)	0.90 (0.82–0.98)	0.89 (0.81–0.97)
≥50	≥50	45,358.6	699	15.4	0.88 (0.81–0.97)	0.82 (0.75–0.90)	0.80 (0.74–0.88)	0.82 (0.76–0.90)

^a^Estimated using Cox proportional hazard models. The multivariable model 1 was adjusted for age, sex, centre, year of screening examination, alcohol consumption, smoking, physical activity, total energy intake, education level, and BMI; model 2: model 1 plus adjustment for medication for hyperlipidaemia, medication for diabetes, multivitamin supplement, vitamin D supplement, and calcium supplement. ^b^Estimated using Cox proportional hazard models with categories of serum 25(OH)D levels, smoking, alcohol consumption, physical activity, total energy intake, BMI, medication for hyperlipidaemia, medication for hyperlipidaemia, medication for diabetes, multivitamin supplement, vitamin D supplement, and calcium supplement as time-dependent variables and baseline age, centre, year of screening examination, and education level as time-fixed variables.

HR, hazards ratio; LMM, low muscle mass; PY, person-years.

In subgroup analyses (Supplementary Table 4), the association between 25(OH)D level and incident LMM differed with respect to hypertension, insulin resistance, and inflammation status; the association was evident in participants without either homeostasis model assessment of insulin resistance (HOMA-IR) of ≥2.5 or hypertension but was attenuated in those with either insulin resistance or hypertension (*P* for interaction <0.001 and 0.002, respectively). The graded dose-response association between 25(OH)D levels and incident LMM was slightly stronger in those with hsCRP < 1.0 mg/L than in those with hsCRP ≥ 1.0 mg/L (*P* for interaction = 0.018). Otherwise, there were no other significant interactions by subgroup, including the age group (<40 vs ≥40 years). Participants taking vitamin D supplements tended to engage in a healthier lifestyle including physical activity and less smoking (Supplementary Table 5); however, after adjustments for physical activity and smoking status, there was an independent and inverse association between serum 25(OH)D levels and incident LMM. Additionally, in subgroup analyses, these associations were similarly observed, and there was no significant interaction, by smoking status, alcohol intake, and physical activity.

## Discussion

In this large cohort study of young Korean adults without LMM at baseline, serum 25(OH)D levels were inversely associated with LMM development in a dose-response manner. The protective association between higher serum 25(OH)D levels and decreased LMM incidence was consistently observed irrespective of sex and age. Furthermore, increases in 25(OH)D levels from insufficient levels at baseline to 50 nmol/L at follow-up and adequate 25(OH)D levels over time were associated with lower risk of incident LMM; these associations were independent of factors such as vitamin D supplementations, exercise, BMI, or season of the blood draw.

It has been well documented that vitamin D insufficiency/deficiency is frequently observed in older people with sarcopenia (6, 7). However, as most studies exploring the link between vitamin D and sarcopenia/LMM by far were almost exclusively focused on older adults, the effect of vitamin D on the risk of LMM among younger adults is unknown. There are few cross-sectional studies that have explored the effects of serum vitamin D levels in muscle mass in younger individuals. A study of 667 community-dwelling adults aged 21–97 years showed significant associations between 25(OH)D levels and muscle mass only in participants younger than 65 years (9). Some other studies have also reported the potential benefit of serum vitamin D on muscle mass; however, these studies were undertaken in specific population subgroups (e.g. obese men) (10, 11). To our knowledge, our study is the first cohort study showing that adequate serum 25(OH)D levels confer decreased risk of incident LMM in young and middle-aged individuals without comorbidities. Also, while there is scarce data on the prevalence of sarcopenia or LMM in young populations, a previous report has estimated that, among adults aged 21–59 years, up to 32% have LMM and 7% have sarcopenia, suggesting that it is already prevalent among younger adults (4). Likewise, our findings on the incident rate of LMM (27.1 per 1,000 person-years) further supports the notion that LMM in young adults is no longer an uncommon condition.

In our study, persistently adequate serum 25(OH)D levels over time and increases in serum 25(OH)D levels from being insufficient to sufficient were significantly associated with decreased LMM risk. The effect of time-dependent changes of serum 25(OH)D levels on preserving muscle mass has been uncertain, with a lack of comparable data. Two previous RCTs evaluated the benefit of vitamin D supplementation and changes in vitamin D levels on muscle mass and strength in young and middle-aged individuals, but neither found any significant benefits of vitamin D in improving muscle mass (12, 13). However, it is difficult to directly compare these study results with ours because these trials were underpowered with a sample size <40 and had a very short-term follow-up (12 weeks) in a setting of resistance training, wherein vitamin D was supplemented only as an adjunct intervention. In our large sample of 192,908 healthy participants free of LMM at baseline, we could account for various known confounders, as well as time-dependent variables. Also, the extended follow-up duration of approximately 4 years allowed the extended time frame for us to better observe the development of LMM over time. Although the possibility of residual confounding remains due to unmeasured factors including sun exposure or outdoor physical activity, our findings suggest that improved or persistently adequate serum 25(OH)D status over time may have benefit in reducing the risk of incident LMM.

The present study has several important clinical implications. Earlier onset of sarcopenia has constantly been increasing, especially in developed countries, possibly owing to changes in lifestyle and diets (4), and there is an emerging need for taking a life-course approach to sarcopenia prevention during early years (4, 36). Given the progressive nature of and the seriousness of disability and complications associated with sarcopenia (1), preventing mild LMM may in turn delay further loss of muscle mass, consequently lowering the risk of sarcopenia as well as sarcopenia-related health consequences in later life. In light of this, our findings suggest that the prevention of early unfavourable changes in muscle mass and mild LMM may be achievable in young individuals by maintaining sufficient serum 25(OH)D levels. In addition, the proportion of our study participants with sub-optimal 25(OH)D levels (<50 nmol/L) at baseline (approximately 74%) is considerably higher than that in the United States and Europe (24–40%) (37), although it is comparable to the previously reported national prevalence in the Korean population (38). We assume that a high proportion of white-collar workers in our population who are likely to have less sun exposure may have contributed to the relatively high prevalence of sub-optimal serum 25(OH)D levels. Our findings thus highlight the importance of maintaining adequate serum 25(OH)D levels to reduce the risk of LMM in populations with a high prevalence of low vitamin D status. Large and well-designed intervention trials are necessary to confirm our findings.

The mechanism by which serum 25(OH)D reduces LMM risk is not completely understood, but recent studies confirm that VDR is expressed in skeletal muscle and that a substantial level of signalling via VDR is required for normal muscle growth and muscle mass maintenance (39). In animal studies, VDR knockout mice had small and variable muscle fibres (40); vitamin D deficiency in rats inhibited mammalian target of rapamycin complex 1 (mTORC1) signalling and contributed to decreased protein synthesis in skeletal muscles (41), while VDR overexpression induced muscle hypertrophy (5). In human muscle tissue, VDR expression levels, which decline with age, can be altered using vitamin D supplementation (42), indicating that maintaining adequate 25(OH)D levels could reduce LMM risk. 25(OH)D may also stimulate protein synthesis through mTORC1 signalling; this mechanism may play an important role in muscle hypertrophy and muscle loss prevention (39).

According to our subgroup analyses, the association between serum 25(OH)D and LMM was attenuated in participants with insulin resistance defined as HOMA-IR ≥ 2.5 and/or hypertension. Skeletal muscle is the key tissue responsible for insulin-stimulated glucose disposal and is the major site of peripheral insulin resistance (43). Muscle mass is determined by the balance between protein synthesis and breakdown in the tissue, and particularly in younger people, insulin has a predominant role in inhibiting protein catabolism, thereby preventing muscle atrophy (44). Insulin resistance thus may represent a state of ‘anabolic resistance’ in skeletal muscle, wherein the insulin-mediated suppression of muscle breakdown is inhibited, potentially leading to increased proteolysis that may eventually result in sarcopenia (45). VDRs are also involved in the pathogenesis of insulin resistance (46); a recent report showed that decreased glucose uptake reduced VDR expression in a diabetic mouse model (47). Therefore, a series of these interactive processes may act synergistically to attenuate the effect of vitamin D. The reason for the null association observed in the presence of hypertension is unclear. Hypertension is pathologically related to hyperactivity of the renin-angiotensin system (RAS), and animal studies show the involvement of VDR activation in downregulating RAS (48). High circulating levels of angiotensin II decrease muscle protein homeostasis and accelerate proteolysis, thereby promoting skeletal muscle fibre atrophy (49). Thus, in hypertension, vitamin D metabolism may not compensate for the effects of RAS overactivation. Future studies are warranted to better elucidate the role of insulin resistance and hypertension in the association between 25(OH)D and LMM.

The strengths of our study include the large sample size, carefully standardized clinical examination, imaging, and laboratory procedures and include the assessment of physical activity and other lifestyle factors. Also, the longitudinal cohort study design with repeated measurements of 25(OH)D levels and confounders enabled us to examine temporal and independent associations of serum 25(OH)D status and changes in serum 25(OH)D status over time with LMM risk. We also considered the changes of covariates during follow-up in our time-dependent model, wherein 25(OH)D levels and other covariates were considered time-varying variables.

This study had some limitations. First, we used bioimpedance analysis instead of DEXA, which is the gold standard body composition measurement for assessing muscle mass. DEXA, however, may expose participants to low-level ionizing radiation and is expensive to perform in large cohort studies. Secondly, we did not collect information on variables that could influence the serum 25(OH)D levels such as vitamin D intake via food consumption, details on the amount and frequency of vitamin D supplementation (e.g. dose, frequency, and duration), outdoor activities, or sunlight exposure, or presence of genetic polymorphism. Therefore, the potential for residual confounding remains. Thirdly, the reference values used in our study in defining LMM were derived from the young adults in this study population since there is no available value derived from the representative sample of the Korean population based on bioimpedance analysis. According to the fourth and fifth KNHANES, the cut-off values of 1 s.d. below the mean for DEXA-based SMI of young adults were 32.2 and 29.9%, respectively, for men and 25.6 and 23.5%, respectively, for women (50, 51), which were similar to the cut-off values used in our study (30.2% for men and 26.1% for women). Finally, our study participants represented a relatively young and healthy Korean working population. Although this could be perceived as a limitation, it also represented a strength of our study as relatively few study participants had existing comorbidities that are associated with low serum 25(OH)D levels. Nevertheless, the generalizability of our findings to other populations with comorbidities or different sociodemographic characteristics may be limited.

In conclusion, we demonstrated that serum 25(OH)D levels are inversely associated with LMM risk in young adults. Favourable changes in serum 25(OH)D levels from insufficient to sufficient were associated with reduced LMM risk. Considering the importance of attaining high peak muscle mass during adulthood for sarcopenia prevention, maintaining sufficient serum 25(OH)D levels, which may be easily achieved by sun exposure or vitamin D supplementation, could be an effective primary prevention strategy to slow muscle loss and its associated consequences in later years.

## Supplementary Material

Supplementary Material

## Declaration of interest

The authors declare that there is no conflict of interest that could be perceived as prejudicing the impartiality of this study.

## Funding

This work was supported by SKKU Excellence in Research Award Research Fund, Sungkyunkwan University http://dx.doi.org/10.13039/501100002647, 2020. C D B is supported in part by the Southampton NIHR Biomedical Research Centre (IS-BRC-20004).

## Author contribution statement

Yejin Kim: interpretation of data, drafting and critical revision of the manuscript. Yoosoo Chang: study concept and design, acquisition of data, interpretation of data, and drafting and critical revision of the manuscript. Seungho Ryu: study concept and design, acquisition of data, analysis and interpretation of data, and critical revision of the manuscript. In Young Cho: interpretation of data and critical revision of the manuscript. Min-Jung Kwon: acquisition of data and critical revision of the manuscript. Sarah H Wild: interpretation of data and critical revision of the manuscript. Christopher D Byrne: interpretation of data and critical revision of the manuscript.
