# Supplementary material for: Serum 25-hydroxy vitamin D and the risk of low muscle mass in young and middle-aged Korean adults
Source: Eur J Endocrinol. 2022 Feb 11;186(4):477–87. doi: 10.1530/EJE-21-1229 (PMC8942330; doi:10.1530/EJE-21-1229)
Supplement: Supplementary Material [file supplementary_material.pdf]

SUPPLEMENTARY MATERIAL

Table of Contents

S-1. Methods: exclusion criteria

eTable 1: Estimated mean values (95% confidence interval) and adjusted proportion (95% confidence interval) of baseline characteristics according to changes in serum 25(OH)D levels in participants at baseline (n=131,595)

eTable 2: Development of low muscle mass according to serum 25(OH)D levels among participants at baseline after further adjustment for waist circumference as a continuous variable instead of body mass index (N=192,696)

eTable 3: Development of class II low muscle mass according to serum 25(OH)D levels among participants without class II low muscle mass at baseline (n=222,902)

eTable 4: Hazard ratios (95% CI) of incident low muscle mass according to serum 25(OH)D levels in clinically relevant subgroups (N=192,908)

eTable 5. Estimated mean and adjusted proportions of baseline characteristics by vitamin D supplement among participants (n=192,908)

S-1. Exclusion criteria

A total of 39,656 participants were excluded in a two-step selection process (**Figure 1**) as follows. **Step 1:** Participants with a history of cancer (n=5,143), low muscle mass (LMM) at baseline (n=35,312), or missing information on body mass index (BMI), appendicular skeletal muscle mass, or serum 25(OH)D levels (n=69) were excluded. Some participants met more than one exclusion criterion, and a total of 192,908 participants who did not have LMM were included in an LMM-free cohort. **Step 2:** To evaluate the association between changes in serum 25(OH)D levels and risk of incident LMM, participants who underwent a comprehensive health examination at baseline but had only one subsequent follow-up visit (n=49,809) were further excluded from the original cohort. Additionally, participants with missing data on serum 25(OH)D levels (n=2,588) and those who presented with LMM on the second visit (n=27,750) were excluded. The remaining 131,595 participants were included in the LMM-free cohort, among whom we examined the association of change in serum vitamin D levels with the risk of incident LMM.

eTable 1. Estimated[a] mean values (95% confidence interval) and adjusted[a] proportion (95% confidence interval) of baseline characteristics according to changes in serum 25(OH)D levels in participants at baseline (n = 131,595)

Characteristics	Serum 25(OH)D levels (nmol/L)			
	<50 at visit 1 & <50 at visit 2	≥50 at visit 1 & <50 at visit 2	<50 at visit 1 & ≥50 at visit 2	≥50 at visit 1 & ≥50 at visit 2
Number of participants	73,917	11,741	23,745	22,192
Age (years)	38.4 (38.4-38.4)	39.0 (38.9-39.1)	37.1 (37.1-37.2)	39.7 (39.7-39.8)
Male (%)	52.53 (52.17-52.89)	65.07 (64.21-65.93)	61.26 (60.64-61.88)	72.37 (71.77-72.96)
Alcohol intake (%) [b]	16.97 (16.69-17.24)	21.72 (21.03-22.41)	19.69 (19.20-20.18)	24.73 (24.22-25.24)
Current smoker (%)	17.14 (16.87-17.41)	20.53 (19.87-21.18)	18.21 (17.74-18.67)	21.42 (20.96-21.88)
HEPA (%)	11.97 (11.74-12.21)	16.10 (15.44-16.76)	13.83 (13.39-14.28)	17.30 (16.81-17.80)
Education level (%) [c]	85.90 (85.66-86.15)	86.18 (85.55-86.80)	86.72 (86.28-87.17)	86.25 (85.79-86.71)
History of diabetes (%)	1.75 (1.66-1.85)	1.62 (1.41-1.84)	1.64 (1.47-1.81)	1.94 (1.79-2.10)
History of hypertension (%)	5.74 (5.57-5.91)	5.78 (5.39-6.17)	6.38 (6.06-6.69)	6.49 (6.21-6.77)
History of CVD (%)	0.72 (0.65-0.78)	0.81 (0.66-0.97)	0.99 (0.86-1.12)	0.81 (0.70-0.92)
Anti-lipid medication use (%)	1.79 (1.70-1.89)	1.70 (1.48-1.91)	1.64 (1.47-1.81)	1.72 (1.57-1.86)
Multivitamin supplement (%)	5.59 (5.43-5.76)	12.56 (11.96-13.16)	6.09 (5.78-6.40)	11.97 (11.54-12.40)
Vitamin D supplement (%)	0.44 (0.40-0.49)	1.40 (1.18-1.62)	0.65 (0.54-0.76)	2.37 (2.15-2.59)
Calcium supplement (%)	0.29 (0.26-0.33)	1.01 (0.81-1.20)	0.47 (0.38-0.56)	1.34 (1.17-1.51)
Obesity (%) [d]	19.93 (19.65-20.21)	21.17 (20.50-21.84)	20.87 (20.38-21.35)	21.69 (21.22-22.16)
Body mass index (kg/m^2)	22.6 (22.6-22.6)	22.7 (22.6-22.7)	22.7 (22.6-22.7)	22.7 (22.7-22.8)
SBP (mmHg)	107.2 (107.2-107.3)	107.5 (107.3-107.6)	108.0 (107.8-108.1)	108.2 (108.1-108.3)
DBP (mmHg)	69.4 (69.3-69.4)	69.6 (69.5-69.8)	69.5 (69.4-69.6)	69.9 (69.8-70.0)
Glucose (mg/dl)	93.6 (93.6-93.7)	93.6 (93.4-93.9)	93.9 (93.8-94.1)	93.9 (93.7-94.0)
Total cholesterol (mg/dl)	191.2 (191.0-191.4)	192.7 (192.1-193.2)	190.8 (190.4-191.3)	192.0 (191.5-192.4)
GGT (U/L)	28.0 (27.8-28.3)	30.5 (29.9-31.0)	28.8 (28.4-29.2)	31.3 (30.9-31.7)
ALT (U/L)	21.9 (21.8-22.1)	22.3 (22.0-22.6)	22.1 (21.9-22.4)	22.7 (22.4-22.9)
HOMA-IR	1.42 (1.42-1.43)	1.37 (1.35-1.39)	1.44 (1.43-1.45)	1.39 (1.38-1.40)
hsCRP	0.89 (0.87-0.92)	0.92 (0.86-0.97)	0.94 (0.90-0.98)	0.93 (0.89-0.96)
Total energy intake (kcal/d) [e]	1,477.9 (1,472.6-1,483.1)	1,478.6 (1,465.5-1,491.7)	1,457.6 (1,448.3-1,466.9)	1,468.8 (1,459.0-1,478.6)

[a] Adjusted for age and sex; [b] ≥10 g/day; [c] ≥College graduate; [d] BMI ≥25 kg/m^2; [e] among 88,596 participants with plausible estimated energy intake levels (within three standard deviations from the log-transformed mean energy intake)

Abbreviations: ALT, alanine aminotransferase; CVD, cardiovascular disease; DBP, diastolic blood pressure; GGT, gamma-glutamyltransferase; HDL-C, high-density lipoprotein-cholesterol; HEPA, health-enhancing physically active; hs-CRP, high- sensitivity C-reactive protein; HOMA-IR, homeostasis model assessment of insulin resistance; SBP, systolic blood pressure.

eTable 2. Development of low muscle mass according to serum 25(OH)D levels among participants at baseline after further adjustment for waist circumference as a continuous variable instead of body mass index (n = 192,696)

25(OH)D levels (nmol/L)	Multivariable-adjusted HR[a] (95% CI)
Total	
<25	1.00 (reference)
25–<50	0.92 (0.88-0.95)
50–<75	0.84 (0.80-0.88)
≥75	0.74 (0.68-0.80)
p–trend	<0.001
Women	
<25	1.00 (reference)
25–<50	0.93 (0.88-0.97)
50–<75	0.85 (0.80-0.91)
≥75	0.73 (0.64-0.82)
p–trend	<0.001
Men	
<25	1.00 (reference)
25–<50	0.90 (0.84-0.96)
50–<75	0.82 (0.76-0.88)
≥75	0.73 (0.65-0.82)
p–trend	<0.001

[a] Estimated using Cox proportional hazard models. The multivariable model was adjusted for age, sex (only for total), center, year of screening examination, alcohol consumption, smoking, physical activity, total energy intake, education level, medication for hypertension, medication for diabetes, multivitamin supplement, calcium supplement, season, and waist circumference.

Abbreviations: CI, confidence interval; HR, hazard ratio

eTable 3. Development of class II low muscle mass[a] according to serum 25(OH)D levels among participants without class II low muscle mass at baseline (n = 222,902)

25(OH)D levels (nmol/L)	Person-years (PY)	Incident cases	Incidence density (/ 10^3 PY)	Age-adjusted HR (95% CI)	Multivariable-adjusted HR[b] (95% CI)		HR (95% CI)[c] in a model with time-dependent variables
					Model 1	Model 2	
Total							
<25	20,958.2	486	23.2	1.00 (reference)	1.00 (reference)	1.00 (reference)	1.00 (reference)
25–<50	70,154.7	1,268	18.1	0.71 (0.64-0.78)	0.82 (0.73-0.91)	0.99 (0.88-1.10)	0.83 (0.76-0.90)
50–<75	28,770.3	390	13.6	0.49 (0.43-0.56)	0.62 (0.53-0.71)	0.84 (0.72-0.97)	0.67 (0.60-0.74)
≥75	8,974.9	67	7.5	0.25 (0.19-0.32)	0.36 (0.28-0.47)	0.60 (0.46-0.79)	0.59 (0.50-0.70)
p–trend				<0.001	<0.001	<0.001	<0.001
Women							
<25	15,219.8	353	23.2	1.00 (reference)	1.00 (reference)	1.00 (reference)	1.00 (reference)
25–<50	37,944.3	712	18.8	0.73 (0.64-0.83)	0.86 (0.75-0.98)	1.10 (0.96-1.25)	0.89 (0.80-0.98)
50–<75	13,090.0	199	15.2	0.54 (0.46-0.64)	0.75 (0.62-0.90)	1.08 (0.90-1.30)	0.80 (0.69-0.91)
≥75	4,584.3	38	8.3	0.27 (0.20-0.38)	0.44 (0.31-0.61)	0.79 (0.56-1.12)	0.77 (0.62-0.96)
p–trend				<0.001	<0.001	0.937	0.001
Men							
<25	5,738.4	133	23.2	1.00 (reference)	1.00 (reference)	1.00 (reference)	1.00 (reference)
25–<50	32,210.4	556	17.3	0.70 (0.58-0.85)	0.70 (0.58-0.85)	0.72 (0.59-0.87)	0.65 (0.56-0.77)
50–<75	15,680.3	191	12.2	0.46 (0.37-0.58)	0.46 (0.37-0.57)	0.53 (0.42-0.66)	0.48 (0.40-0.57)
≥75	4,390.6	29	6.6	0.23 (0.16-0.35)	0.26 (0.18-0.40)	0.37 (0.25-0.55)	0.38 (0.29-0.50)
p–trend				<0.001	<0.001	<0.001	<0.001

Note: P <0.001 for the overall interaction between sex and serum 25(OH)D levels for incident low muscle mass (Multivariable-adjusted model 2)

[a] defined as the appendicular skeletal muscle mass divided by body weight of minus two standard deviations below the sex-specific mean for the young reference group

[b] Estimated using Cox proportional hazard models. Multivariable model 1 was adjusted for age, sex (only for total), center, year of screening examination, alcohol consumption, smoking, physical activity, total energy intake, education level, medication for hypertension, medication for diabetes, multivitamin supplement use, calcium supplement use, and season; model 2: model 1 plus adjustment for body mass index

[c] Estimated using Cox proportional hazard models with quintiles of serum 25(OH)D levels, smoking, alcohol consumption, physical activity, total energy intake, medication for hypertension, medication for diabetes, multivitamin supplement use, calcium supplement use, season, and body mass index as time-dependent variables and baseline age, sex (only for total), center, year of screening examination, and education level as time-fixed variables

Abbreviations: CI, confidence interval; HR, hazards ratio; PY, person-years

eTable 4. Hazard ratios[a] (95% CI) of incident low muscle mass according to serum 25(OH)D levels in clinically relevant subgroups (n = 192,908)

Subgroup	Serum 25(OH)D levels (nmol/L)				*P* for interaction
	<25	25–<50	50–<75	≥75	
**Age**					0.175
<40 years (n=125,850)	Reference	0.92 (0.88-0.97)	0.84 (0.79-0.90)	0.71 (0.63-0.79)	
≥40 years (n=67,058)	Reference	0.95 (0.90-1.01)	0.85 (0.79-0.92)	0.84 (0.75-0.94)	
**Current smoking**					0.209
No (n=153,682)	Reference	0.92 (0.89-0.96)	0.85 (0.81-0.90)	0.76 (0.69-0.83)	
Yes (n=32,420)	Reference	1.04 (0.92-1.18)	0.91 (0.79-1.03)	0.87 (0.72-1.04)	
**Alcohol intake**					0.312
<20 g/day (n=148,520)	Reference	0.93 (0.90-0.98)	0.86 (0.81-0.91)	0.80 (0.72-0.88)	
≥20 g/day (n=36,544)	Reference	0.90 (0.81-1.01)	0.78 (0.69-0.88)	0.71 (0.60-0.84)	
**HEPA**					0.333
No (n=163,448)	Reference	0.94 (0.90-0.98)	0.84 (0.80-0.89)	0.80 (0.73-0.88)	
Yes (n=27,274)	Reference	0.96 (0.86-1.08)	0.91 (0.80-1.03)	0.72 (0.60-0.88)	
**Body mass index**					0.879
<25 kg/m^2 (n=151,710)	Reference	0.94 (0.89-0.98)	0.83 (0.78-0.88)	0.72 (0.64-0.80)	
≥25 kg/m^2 (n=41,198)	Reference	0.91 (0.85-0.97)	0.81 (0.75-0.88)	0.70 (0.62-0.80)	
**Hypertension**					0.002
No (n=177,568)	Reference	0.92 (0.88-0.96)	0.83 (0.79-0.87)	0.73 (0.66-0.79)	
Yes (n=15,329)	Reference	1.09 (0.95-1.24)	1.02 (0.88-1.18)	1.10 (0.90-1.36)	
**Diabetes**					0.484
No (n=188,312)	Reference	0.93 (0.90-0.97)	0.84 (0.80-0.88)	0.76 (0.70-0.83)	
Yes (n=4,595)	Reference	0.99 (0.77-1.28)	1.01 (0.77-1.34)	0.90 (0.60-1.35)	
**HOMA-IR**					<0.001
<2.5 (n=173,293)	Reference	0.90 (0.87-0.94)	0.80 (0.76-0.85)	0.74 (0.68-0.81)	
≥2.5 (n=18,882)	Reference	1.10 (0.99-1.21)	1.07 (0.96-1.21)	0.92 (0.75-1.14)	
**hsCRP**					0.018
<1.0 mg/L (n=157,230)	Reference	0.91 (0.87-0.95)	0.81 (0.77-0.86)	0.76 (0.69-0.84)	
≥1.0 mg/L (n=35,491)	Reference	1.01 (0.93-1.10)	0.96 (0.87-1.05)	0.79 (0.68-0.93)	

Estimated using Cox proportional hazard models. The multivariable model was adjusted for age, sex, center, year of screening examination, alcohol consumption, smoking, physical activity, total energy intake, education level, medication for hypertension, medication for diabetes, multivitamin supplement use, calcium supplement use, season, and body mass index

Abbreviations: CI, confidence interval; HOMA-IR, homeostasis model assessment of insulin resistance; hsCRP, high-sensitivity C-reactive protein

eTable 5. Estimated[a] mean and adjusted[a] proportions of baseline characteristics by vitamin D supplement among participants (n = 192,908)

**Characteristics**	Vitamin D supplement (%)		***p*-Trend**
	No use	Use	
Number of participants	190,999	1,909	
Age (years)	37.8 (37.8-37.8)	41.8 (41.5-42.2)	<0.001
Male (%)	55.77 (55.5-56.0)	28.7 (26.8-30.7)	<0.001
Alcohol intake (%) [b]	19.7 (19.6-19.9)	20.6 (18.5-22.7)	0.429
Current smoker (%)	17.4 (17.3-17.6)	15.4 (13.5-17.4)	0.051
HEPA (%)	14.3 (14.1-14.4)	18.7 (16.9-20.4)	<0.001
Education level (%) [c]	84.7 (84.5-84.8)	86.1 (84.7-87.5)	0.050
History of diabetes (%)	1.79 (1.74-1.85)	1.83 (1.34-2.32)	0.895
History of hypertension (%)	6.05 (5.94-5.15)	7.90 (6.73-9.07)	0.001
History of CVD (%)	0.82 (0.78-0.86)	1.21 (0.81-1.61)	0.024
Anti-lipid medication use (%)	1.79 (1.74-1.86)	3.08 (2.46-3.70)	<0.001
Multivitamin supplement (%)	6.73 (6.62-6.84)	38.41 (36.26-40.56)	<0.001
Calcium supplement (%)	0.45 (0.42-0.48)	6.80 (5.89-7.71)	<0.001
Obesity (%) [d]	21.4 (21.2-21.5)	19.3 (17.3-21.3)	0.045
Body mass index (kg/m^2)	22.7 (22.7-22.7)	22.6 (22.5-22.7)	0.064
SBP (mmHg)	107.5 (107.5-107.6)	107.8 (107.4-108.3)	0.177
DBP (mmHg)	69.3 (69.2-69.3)	69.0 (68.6-69.3)	0.121
Glucose (mg/dL)	93.5 (93.4-93.5)	93.1 (92.6-93.6)	0.158
Total cholesterol (mg/dL)	190.7 (160.6-190.9)	191.2 (189.7-192.6)	0.577
GGT (U/L)	28.4 (28.2-28.5)	28.1 (26.6-29.6)	0.742
ALT (U/L)	22.0 (21.9-22.1)	23.4 (22.6-24.2)	<0.001
HOMA-IR	1.43 (1.43-1.44)	1.39 (1.35-1.43)	0.005
hsCRP (mg/L)	0.91 (0.90-0.93)	0.96 (0.83-1.10)	0.399
Total energy intake (kcal/d)[e, f]	1,442 (1,439-1,445)	1,519 (1,486-1,553)	<0.001

[a] Adjusted for age and sex; [b] ≥20 g/day; [c] ≥College graduate; [d] Body mass index ≥25 kg/m^2; [e] among 132,466 participants with plausible estimated energy intake levels (within three standard deviations from the log-transformed mean energy intake); [f] 1 kcal equals to 4,185.8 J

Abbreviations: ALT, alanine aminotransferase; CVD, cardiovascular disease; DBP, diastolic blood pressure; GGT, gamma-glutamyltransferase; HEPA, health-enhancing physically active; hsCRP, high-sensitivity C-reactive protein; HOMA-IR, homeostasis model assessment of insulin resistance; SBP, systolic blood pressure
